# Conscious birds

**DOI:** 10.1098/rstb.2024.0308

**Published:** 2025-11-13

**Authors:** Gianmarco Maldarelli, Onur Güntürkün

**Affiliations:** ^1^ Department of Biopsychology, Institute of Cognitive Neuroscience, Faculty of Psychology, Ruhr University Bochum, Bochum, Nordrhein-Westfalen, Germany

**Keywords:** birds, prefrontal cortex, lateralization, avian, pigeon

## Abstract

In this article, we start from the assumption that consciousness is not the ultimate triumph of human evolution but rather represents a more basic cognitive process, possibly shared with other animal phyla. In this article, we show that there is growing evidence that (i) birds have sensory and self-awareness, and (ii) they also have the neural architecture that may be necessary for this. We present behavioural studies and recent neurobiological data and discuss them in relation to three major theories of consciousness: the Global Neural Workspace Theory (GNWT), the Recurrent Processing Theory (RPT) and the Integrated Information Theory. Although the findings so far do not allow for a strong conclusion, the neurophysiological and anatomical features of the avian brain seem to align with the prerequisites of the GNWT and RPT to host consciousness.

This article is part of the theme issue ‘Evolutionary functions of consciousness’.

## 1. Introduction

Just two decades ago, neuroscientists were highly sceptical about the possibility of consciousness in non-human animals. Today, the tables have turned, and comparative cognitive neuroscientists increasingly consider it plausible that different species could possess consciousness. This change has been prompted by theoretical advances and novel neuroscientific experiments. As a result, consciousness in non-human animals is not only hotly debated (e.g. [1–3]), but sometimes also taken for granted, at least in great apes and some monkey species [4,5]. Therefore, the scientific community has nowadays been discussing the presence of consciousness in animals such as insects and fishes [6,7], while also proposing alternative approaches to explore animal consciousness [8].

One of the current conundrums of consciousness concerns its function(s). While some have asserted that it has no function and no causal role in cognitive processes, other authors have suggested that consciousness enables various behavioural and mental functions that an unconscious organism could not recruit (for a review, see [9]). Because evolution can only operate by selecting an animal’s behavioural output, we assume that theories of consciousness must account for both its neural basis and its behavioural implications. When looking at their behavioural repertoire and cognitive abilities, birds have proved to be much smarter than previously thought [10–15]. However, are they therefore conscious? This discussion has started, and some recent interesting findings have stimulated the debate on avian consciousness [16–19]. In this review, we will show the most recent discoveries in the field of avian consciousness in terms of sensory- and self-awareness, and we will argue that evidence for that is already emerging. Besides that, many theories of consciousness have been proposed in the last decades to put forward the neural mechanisms involved in subjective experience (for a review, see [20,21]). However, they have been mainly designed and tested on humans or non-human primates. Thus, we will discuss whether the avian brain could also meet their requirements. To do so, we will take into account three major theories of consciousness, i.e. the Global Neural Workspace Theory (GNWT), the Recurrent Processing Theory (RPT) and the Integrated Information Theory (IIT).

These three theories are based on different assumptions and neural mechanisms and state different predictions. The GNWT holds that consciousness arises when information becomes globally available through a nonlinear ‘ignition’ across a distributed network of long-range neurons, particularly involving parietal and prefrontal areas [22,23]. Conscious content becomes available when it is selected, amplified and broadcast across brain areas, enabling reportability, flexible routing and integration of features [23,24]. Experimental findings support key predictions, such as spontaneous ignition and the decoding of conscious content from prefrontal neural activity, including abstract and sensory information [5,25].

The RPT posits that conscious visual perception arises from local recurrent interactions within the visual cortex [26,27]. Empirical findings show that feedforward processing, while capable of activating both low- and high-level areas, does not correlate with consciousness, whereas recurrent activity is consistently associated with conscious experience [28–30]. RPT advocates for consciousness as an independent process, orthogonal to cognition and attention [31].

The IIT explains consciousness by starting from the phenomenology of subjective experience and its essential properties [32]. It proposes that consciousness corresponds to the cause–effect structure (Φ-structure) of a physical substrate, defined by how the parts of a system influence each other [33]. Consciousness is quantified by Φ, while the specific quality of experience depends on the structure of causal relationships within the system [32]. IIT suggests that regions like the posterior cortex support high Φ because of their integrated architecture [34], whereas structures like the cerebellum do not, owing to their modular organization. In the following, we will first show studies of sensory awareness in birds and then discuss whether their neurophysiology and neuroanatomy fit with the assumptions and predictions of the mentioned theories. Last, we will discuss studies about avian self-awareness.

## 2. Sensory awareness

Our brain is constantly bombarded with incoming information that must be filtered according to its relevance through attentional mechanisms. The perceptual result is a subjective representation of only a small portion of the sensory information captured by the sensory organs. This piece of information accesses consciousness and can thus be reported. By contrast, the rest is detoured to subconscious processes, where they are still able to modify behaviour, but without our overt awareness [35,36]. A particular case is represented by ambiguous stimuli, which can be interpreted in several alternative ways (e.g. the famous Necker cube). These stimuli represent a challenge for the organism since it is unclear how to respond to them properly. Most subjects that look at an ambiguous stimulus experience a repeated switch between two perceptions, a phenomenon called perceptual rivalry [37]. The scientific beauty of this phenomenon is that the stimulus itself stays physically identical, while its subjective perception changes between two interpretations. This implies that physical stimuli are not simply represented as they are but are interpreted according to our expectations. This was first proposed by Hermann von Helmholtz when he discovered that humans tested under monocular conditions ‘saw’ letters in their retinal blind spot that seemed to make sense, given the surrounding text ([38], p. 579). These and similar observations led von Helmholtz to conclude that perceiving is an active sensory process in which observations arise from a mixture of perceptions and expectations. The perceptual switch effect, therefore, occurs since some objects offer two different perceptual interpretations, resulting in periodic alternations of perceptions. The neural correlates of this phenomenon in humans seem to engage not only posterior visual areas but also a distributed frontoparietal network, including regions in the superior parietal lobule and prefrontal cortex (PFC) [39–41]. These neuroimaging findings suggest that bistable perception is governed by an active interplay between bottom-up sensory processing and top-down inferential mechanisms across hierarchical brain systems.

This effect seems to be widespread across animal species, from primates to insects [42], and is also present in birds, as demonstrated by Vetter *et al.* [43]. In that study, pigeons were presented with a pattern of flashing light spots that elicit apparent movement, either horizontally or vertically. By reporting the subjectively perceived movement, pigeons showed a clear response alternation over time, suggesting a bistable perception. These results indicate that also the avian visual system can perceive several perceptual interpretations that compete to access consciousness, and at a certain point in time, only one of them is the ‘winner’.

**Figure 1.**
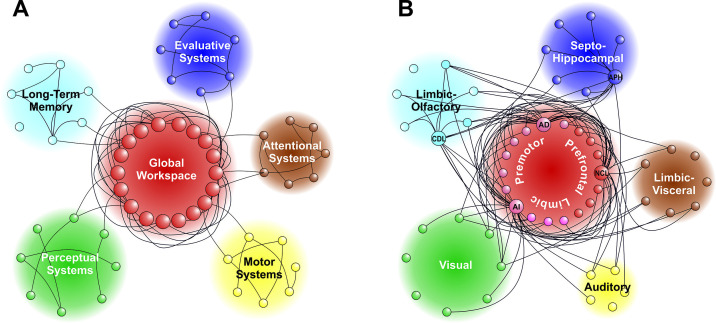
Global Neuronal Workspace Theory (GNWT) and the avian connectome. (A) The GNWT requires local and specialized neuronal modules (small circles grouped by function in the external networks) linked to a central, highly interconnected global workspace. A strong activity in any of the specialized modules can gain access to information flow in the core global workspace and ignite an activity that spreads over most of the network. This process is thought to accompany a conscious representation of the event that caused the ignition. The GNWT depicted here is based on [44] and faithfully redrawn to overlap in colours and style with the pigeon telencephalic connectome. (B) The telencephalic connectome of the pigeon forebrain as analysed with graph theoretical approaches, showing exclusively the connections to and from the hub nodes. Note that few integrative hubs (big circles)—namely nidopallium caudolaterale (NCL), arcopallium dorsale (AD), arcopallium intermedium (AI), area parahippocampalis (APH) and area corticoidea dorsolateralis (CDL)—are densely interconnected to each other as well as to the localized modules (small circles). This represents a potential substrate for a global workspace (figure modified from [45], while reproducing all connections and their allocated colours).

However, what are the neural substrates underlying the conscious and unconscious streams of perception? As already mentioned in §1, in recent times, several theories of consciousness have been proposed to offer an answer [20,21]. Here, we present the neural correlates posited by the GNWT, the RPT and the IIT. In short, the GNWT posits that the constitutive neuroanatomical elements of the conscious brain are (i) localized and specialized modules that are constituted by cortical areas that process a specific type of information, such as perception, motor execution, etc.; and (ii) a structural core of hubs that, through feedback and feedforward connections, distribute and broadcast the information processed by the specific modules and reciprocally communicate with the wider neural network (figure 1A). Given this configuration, the conscious perception of a given stimulus takes place in two main steps: (i) the perceptual input is first processed by locally specialized perceptual modules of the neural network (e.g. the image of a painting processed by visual cortical areas). If this perceptual input gains momentum, (ii) its information can be globally broadcast across the connectome to integration hubs such as the PFC [23,46]. If this broadcasting from the local modules to the global workspace takes place (also known as ‘ignition’), the wide availability of the modules’ information across the network starts a conscious experience. Although this is still a matter of debate, neurophysiological evidence for this theory has already been shown in non-human primates [4,5].

According to RPT, consciousness is constituted by recurrent processing in the sensory pathways [26,27]. In general, two requirements should be met for a nervous system to compute conscious perception. Anatomically, there should be feedforward and feedback connections along the sensory pathways and their links to parieto-frontal structures. Secondly, at a neurophysiological level, these neurons should exhibit recurrent bottom-up and top-down signalling during perception. This is based on the empirical evidence that visual processing starts with an unconscious ‘feedforward sweep’ along the visual areas, followed by feedback interactions that accompany subjective experience [28]. Importantly, this theory discriminates between two stages of consciousness. The first one is a basic subjective experience of the stimulus (‘seeing’), when the recurrent processing is limited to the sensory areas. A deeper conscious experience (‘knowing’) that gives access to report and working memory takes place when the recurrent processing is also extended to the prefrontal areas [21].

According to IIT, a substrate (e.g. a brain area) involved in consciousness must have a maximum intrinsic cause–effect power [33]. This property is represented by a value (Φ) that measures how much information is generated by a system compared to its parts when considered independently. Given the challenge of performing this calculation in complex biological systems such as brains, a good substitute is the Perturbational Complexity Index (PCI), which measures the algorithmic (Lempel-Ziv) complexity of brain responses to transcranial magnetic stimulation [47].

The requirements of these three theories can be confronted with the two studies in birds showing neurophysiological data related to sensory awareness [17,19]. In both studies, crows performed a delayed stimulus detection task: sometimes the stimuli were presented, other times not, and the crows reported whether they saw them or not. The stimuli had a variable level of intensity. So, when they were at the subjective sensitivity threshold, they would be detected in only about half of the cases. Single unit activity of the nidopallium caudolaterale (NCL)—the functional analogue of the mammalian PFC [48,49]—correlated with the subjective report (i.e. when the stimulus was reported as ‘seen’), regardless of the actual presence or absence of the stimulus. The neurons were also silent when the crow reported the stimulus as ‘not seen’. Because the NCL activity predicted the subjective report rather than the visual input, this study represents, to our knowledge, the first evidence of an avian brain area linked to subjective experience.

Moreover, even the absence of a stimulus might be coded in the NCL when it is task relevant. Using the same task described above [19], the authors showed two neural subpopulations in the crow NCL: one encoding for the presence of the cue and one for its absence. The absence of the stimulus was task relevant, as the crows were trained to report that as well. Thus, it became a salient behavioural category through learning.

The current neurophysiological data [17] do not allow for clear-cut interpretations in favour of any particular theory. In fact, the results of Nieder and colleagues could be explained by any of these theories.

The GNWT would interpret the NCL activation as a correlate of sensory awareness. However, the second study [19] can potentially expand or challenge the theoretical framework of the GNWT, because not only did the visual input elicit the ignition of conscious perception but also its absence, at least when reporting the absence was part of the task.

The RPT could qualify the activity in NCL as a conscious percept accompanied by higher-order processes (such as subjective report and working memory) only if recurrent processing takes place (‘knowing’). However, local recurrent processing within the visual areas would already be sufficient for a non-reportable percept (‘seeing’). Unless the contribution of recurrent processes is tested experimentally by using optogenetic tools, this would not be proof. However, there is strong evidence from other studies that information propagated through feedback and feedforward projections during tasks involving perceptual events alters the dynamics of the cellular population [50–53]. Therefore, the neurophysiological requirements of RPT seem to be met.

Finally, the IIT would consider the NCL activation as task-related processing. A neural process would be considered a mechanism of consciousness only if Φ (or its proxy) is sufficiently high to indicate information integration. This approach has not yet been tested in birds, and the current data make it difficult to make reasonable predictions. Therefore, measuring PCI in birds for different global states of consciousness (e.g. awake, during non-rapid eye movement (NREM) sleep, anaesthesia), as it has already been done in rodents [54], would be an important enterprise for future studies.

## 3. Consciousness and the avian neuroanatomy

Classically, the emergence of consciousness is tied to the presence of the highly organized cortex of mammals. Most cortical tissue is isocortical and, therefore, rather uniform across the cortical expanse. Essentially, it consists of a columnar and laminar organization with orthogonally organized fibres running in radial and tangential directions. The radial fibres form repetitive canonical circuits as computational units that process input–output connections and tangentially link all radially incoming information to other isocortical columns. The avian brain, on the other hand, was seen as having a nuclear or cluster conformation with these clusters appearing homogeneous like thalamic nuclei. Nowhere was a laminar or columnar pattern visible. However, a cortex-like organization has recently been discovered in the sensory areas of the avian pallium [55]. This was achieved by a combination of three-dimensional polarized light imaging (3D-PLI) and local tracing techniques.

3D-PLI can identify the orientation of individual axons and render them into false-colour orientation maps, revealing the organization of axonal pathways. This technique showed that the avian sensory pallium consists of layers and columns combined with horizontal and vertical fibres and iteratively repeated canonical circuits. Thus, although birds have a cortex-like structure, it is restricted to the sensory areas of the pallium. By contrast, the associative NCL involved in sensory awareness processing lacks such cortical organization and instead has a nuclear organization. This could imply that a cortex-like structure provides computational advantages for sensory processing, whereas conscious processing is possible without it. The advantage of lamination for sensory processing may arise from the fact that sensory input arrives with its original spatial organization (e.g., a retinotopic map). Preserving this computationally advantageous input requires a neural two-dimensional representation onto which the topographic input is projected. If each point of this two-dimensional area must be analysed by specialized neural circuits for, e.g. edges, luminance, colour, etc., several stacked two-dimensional layers emerge. This is exactly the three-dimensional design of a cortex with its layers and its orthogonally oriented canonical circuits. If birds have consciousness and avian consciousness arises (at least in part) by computations within NCL, consciousness should, in principle, be able to emerge from nuclear structures. Thus, the neural fundaments of consciousness offer more degrees of freedom than anticipated and can be successfully implemented both within the cortical mammalian PFC (and possibly beyond) and the nuclear avian NCL. However, the main feature required for the emergence of consciousness in both mammalian and avian brains might be something else: the brain connectome.

As mentioned earlier, according to the GNWT, the main prerequisite for a conscious brain is a connectome with a core globally connected to smaller local modules (figure 1A). Such a network has already been demonstrated in the mammalian brain [56–59]. Despite the different neuronal architectures between bird and mammal brains, both exhibit similar connectivity. In particular, the PFC of mammals and the NCL of birds act as important integration hubs and are strongly connected to local processing modules ([45]; figure 1B). Thus, this macroscale network appears to be conserved across distant animal phyla. Which brain area could then take over the role of global workspace in other taxa? Are these areas a product of common descent or convergent evolution? For example, Zacks *et al*. [60] argued that, according to comparative neuroanatomy in the main lineages of jawed fishes, the global workspace in early vertebrates may have been the hippocampal homologue. Subsequently, throughout mammalian and avian evolution, the PFC and caudate nucleus may have taken over the role of integration hub of the global workspace, while the hippocampus retained the function of memory formation. The evolutionary advantages would be faster memory formation and higher processing capacity [60].

According to RPT, anatomically, there should be feedforward and feedback connections along the sensory pathways and their links to parieto-frontal structures. The avian visual system indeed exhibits recurrent connectivity throughout its isocortical areas [55]. This is also true for the NCL [61,62] as well as further pallial structures [45,62].

According to IIT, the involvement of a substrate in consciousness relies on its structure. This is quantified by implementing a mathematical framework that unfolds the cause–effect power of the given substrate. This physical structure can then account for all the properties of consciousness. The possible empirical evidence of a brain area with high cause–effect power would be a ‘grid-like’ structure, as observed in the mouse cortex [34]. The main empirical evidence for grid-like structures in the mammalian brain lies in the presence of cell type-specific clusters—known as microcolumns—arranged in a lattice formation within cortical layer 5 [21,34]. These microcolumns form a hexagonal mosaic that extends across the central and posterior regions of the cortex, creating a recurring pattern of information processing that resembles a two-dimensional grid. At this point, it is important to say that currently a causal link between a cortical lattice formation and consciousness has not been shown. Thus, this relationship is at present hypothetical.

A lattice-like structure has not yet been identified in the avian brain, but otherwise, the sensory pallium of birds shares a cortex-like architecture with canonical microcircuits that are interconnected via horizontal projections [55]. Nevertheless, it remains unclear what kind of network properties emerge from cell type-specific columns in birds. To establish the presence of a substrate for consciousness as defined by IIT, it might be essential to investigate whether a comparable two-dimensional-grid system also exists in the cortex-like pallium of avian species.

In summary, according to GNWT, the neurophysiological data suggest that NCL activation in birds correlates with sensory awareness. Anatomically, avian brains show similar connectivity patterns to mammals, with key integration hubs like the NCL, indicating that GNWT’s requirements for a conscious brain are largely met. Regarding RPT, the neurophysiological evidence supports the idea that recurrent processing could contribute to consciousness, though it remains unproven without experimental validation. Anatomically, birds’ visual system and NCL exhibit recurrent connectivity, fulfilling RPT’s anatomical requirements. With respect to IIT, a measure of the integrated information at both a physiological level (like PCI) and an anatomical level (a ‘grid-like’ structure) has not yet been observed. Therefore, the physiological and anatomical requirements for IIT are not met (yet), although the cortex-like modularity of the sensory avian pallium is the necessary prerequisite to host a grid-like architecture. Many more studies are needed before stronger conclusions can be drawn about the neural basis of consciousness in birds. As for the GNWT, we do not yet know the difference between conscious and unconscious perception at the neural network level beyond the NCL. If GNWT is true, we would expect constant activation in the primary and associative visual areas under both conditions, but the NCL should additionally be activated when the stimulus is consciously perceived. Furthermore, milestone paradigms of human consciousness (such as binocular rivalry and flash suppression) have not yet been tested in birds. Therefore, it is still unclear whether birds can experience binocular rivalry and, if so, how this phenomenon can be implemented in a brain with a very different arrangement of visual information than in mammals. For RPT, switching on and off recurrent processes would be required to investigate conscious perception. For IIT, the PCI could be measured to clarify the matter. Such future studies would certainly pave the way for a deeper understanding of the minimum requirements for subjective experiences in non-human animals like birds.

## 4. Self-awareness

Another aspect of consciousness is the ability to be aware of oneself. In humans—but most likely in other species as well—this encompasses several levels, such as the distinction between self- and externally controlled movements, the feeling of how others perceive us, and self-recognition [63]. In animal research, the classic litmus test for assessing self-awareness is the mirror self-recognition (MSR) test, i.e. the recognition of one’s own mirror image. In infants and non-speaking animals, this is usually tested using the marking test, which consists of a two-step procedure. In the first step, the animal is habituated to a mirror. Usually, most individuals initially show social reactions to their mirror image. The crucial aspect is whether this behaviour stops and is replaced by conditional and self-related behaviours, such as using the mirror to see otherwise invisible parts of one’s own body (e.g. the forehead). In the second step, a marker is attached to a part of the subject’s body that is not directly visible (e.g. on the throat or forehead).

After the confrontation with the mirror, the crucial test is whether the animal reaches for the marked body part to remove or examine it [64]. If it does, the species passes the test and can be classified as self-aware. Otherwise, it is assumed to be unaware of itself. The simplicity of the procedure and the clear dichotomous result made this task popular, and it was tested on a wide range of animal species. As a result, a handful of animal species were found to be self-aware (chimpanzees: [65]; orangutans: [66]; dolphins: [67,68]; elephants: [69]; cleaner wrasse: [70,71]; some avian species: see below). However, this methodology has been heavily criticized owing to ecological constraints and the possibility of high rates of false negatives [70,72,73]. Even in humans, the culture in which a child is raised has a remarkable influence on the ability to pass the marking test: one study showed that only 2 out of 82 18- to 72-month-old Kenyan children passed this test, compared to a success rate of 60–85% among Western middle-class children at 20 months of age [74]. Therefore, in recent years, other ecologically more meaningful paradigms based on the MSR have been tested, which have yielded interesting results, particularly in the field of bird research.

In recent decades, many bird species have been subjected to the MSR test (for an overview, see [54]). Some of them have attempted to demonstrate the ability of corvids to recognize themselves after mirror exposure. So far, only magpies ([16] but see [75]) and Indian house crows [76] exhibited a clear mark-directed behaviour; many other corvid species failed the test [77–81]. However, before concluding that many corvid species do not have self-awareness, we should entertain the option that this task lacks some ecological and methodological validity [82]. At a closer look, corvid species such as carrion crows and common ravens show interest in the reflected image when in front of the mirror and show self-directed behaviour (e.g. autopreening, scratching, shaking or bristling) or display contingency checking [79,81]. However, they fail in the final mark test. When it comes to human infants, these behaviours would be indicative of an intermediate step of self-awareness [63].

By using an ecological approach, further surprising results can be obtained in pigeons and chickens [18,83]. Although pigeons can be trained to perform a behaviour similar to self-recognition, they do not spontaneously show it when placed in front of a mirror [84]. However, they are able to discriminate between live and pre-recorded videos of themselves [85]. Furthermore, their reaction rate correlated well with the temporal discrepancy between the subject’s movements and the corresponding video feedback when tested with self-filming videos with different delays (between 1 and 7 s). This suggests that pigeons can recognize the temporal proximity between their behaviour and the corresponding sensory feedback.

**Figure 2.**
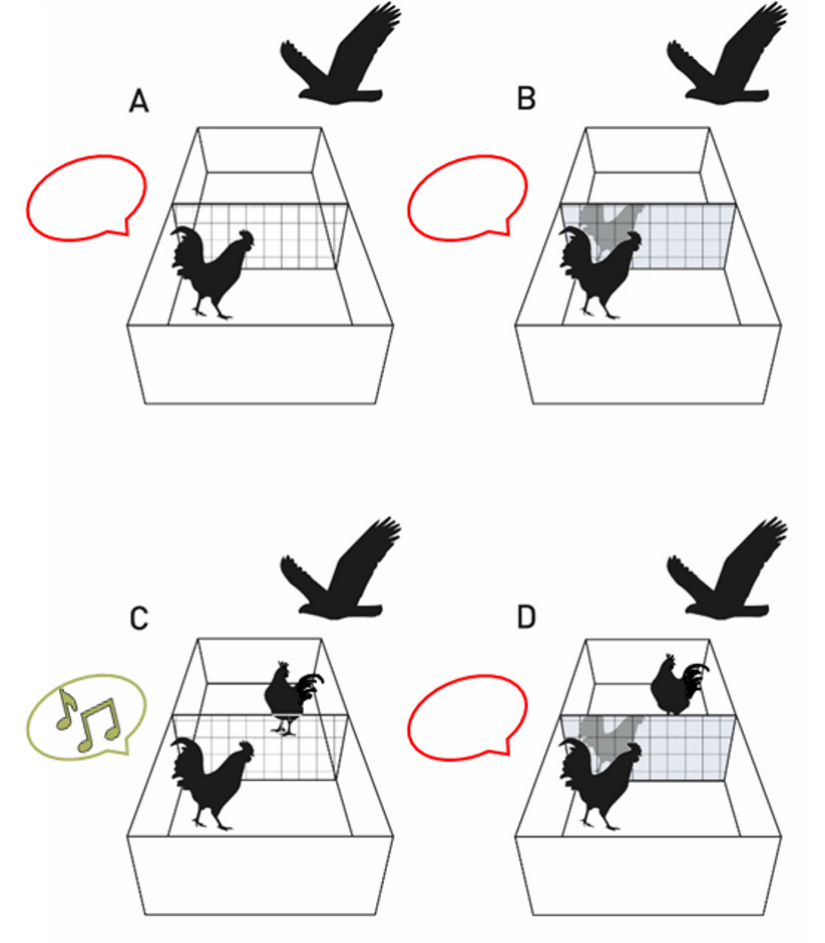
Experimental conditions of the ‘mirror-audience test’ in roosters The tested rooster was placed in an arena divided by a transparent glass (A,C) or a mirror (B,D). The warning vocalizations were recorded while the shadow of a bird of prey was projected onto the ceiling. As expected, the rooster did not vocalize when alone (A) and warned the conspecific when visible (C). Interestingly, the rooster did not warn its own reflected image (B), even when a conspecific was present behind the mirror (D). This figure is reused from Hillemacher *et al*. [18] under the terms of the Creative Commons Attribution (CC BY) license.

Moreover, pigeons appear to treat their reflected image differently than a conspecific: when confronted with a potential competitor for food resources behind a transparent panel, they are more active and display stress-related behaviour more often than when they see their mirrored image [83]. Thus, under conditions of food competition, their mirror image is not perceived as another pigeon. Another study developed an ecologically meaningful mirror test paradigm for roosters—called the ‘mirror-audience test’—which made use of the fact that when roosters detect a predator, they usually warn the flock about the threat [18]. Most importantly, they stay silent when alone. Thus, under threat conditions, roosters can discriminate between being alone or in the company of others. In this experiment, the set-up consisted of an arena divided into two compartments by a transparent panel or a mirror. The tested rooster was placed in one compartment, and its behaviour was observed while the moving shadow of a bird of prey was occasionally projected onto the ceiling. In some trials, a conspecific was also present in the other compartment, while at other times, the tested rooster was alone. As expected, in the conditions with the transparent glass, the roosters vocalized to warn the visible conspecific and stayed silent when they were alone (figure 2A,C). However, in the mirror conditions, the roosters did not warn their reflected image, even when a conspecific was on the other side, covered by the mirror (figure 2B,D). This indicates that, although chickens fail the mark test, they can still discriminate between their image and another conspecific. Both studies indicate that pigeons and chickens do not treat their image as a typical conspecific, probably because they detect the synchronicity between self-movements and the mirrored image. Because they do not pass the mark test, we might assume they mistake their mirror image for an odd stranger rather than themselves. However, that is not what they do: they show strong signs of knowing that their mirror image is not another individual of their species. At least in these two species, this cognitive ability is situationally embedded and can be activated in the right situation, such as when confronted with a predator.

## 5. Conclusion

Behavioural and neurophysiological data suggest that some bird species are aware of external stimuli and recognize themselves to varying degrees in the mirror. Despite large differences in the general brain organization between birds and mammals, the requirements for consciousness as outlined in GNWT and RPT seem to be mostly met. However, evidence of consciousness according to the IIT has not yet been shown. Furthermore, studies on self-recognition in birds provide further behavioural evidence for the limitations of current behavioural paradigms (i.e. the mark test) for evaluating signs of self-awareness. These findings could provide insights into the bigger picture of the evolutionary roots of consciousness. For example, self-awareness appears to be adapted to the ecological constraints of the species, as can be seen from the mirror experiments. Moreover, consciousness should not be deemed as an ‘all-or-nothing’ cognitive function but rather as a graded [63,73] and multi-dimensional process [2]. The presented results add to the growing body of evidence that consciousness may be present in many parts of the animal kingdom, across species that are phylogenetically distant from each other and have remarkably different brain structures. It is certainly too early to speculate whether this is the result of convergent evolution or results from a distant common ancestor, but if so many animals with such diverse brain organisations are conscious, it becomes increasingly likely that consciousness adds cognitive abilities that are evolutionarily successful.

## Data Availability

This article has no additional data.
